# Supplementary material for: Hematopoietic Prostaglandin D Synthase Is Increased in Mast Cells and Pericytes in Autopsy Myocardial Specimens from Patients with Duchenne Muscular Dystrophy
Source: Int J Mol Sci. 2024 Feb 3;25(3):1846. doi: 10.3390/ijms25031846 (PMC10855661; doi:10.3390/ijms25031846)
Supplement: Supplementary file 1 [file ijms-25-01846-s001.zip › ijms-2821815-supplementary.pdf]

Supplementary Table S1. Primer sets for Quantitative RT-PCR analysis of gene expression.

Gene	Sequence	
HPGDS	Forward	5′-TCTTCACCAGAGCCTAGCAAT-3′
	Reverse	5′-AAGTCTGCCCAAGTTACAGAGTT-3′
COX1	Forward	5′-GGAGTTCTACCCTGGACTGC-3′
	Reverse	5′-AGACCAGCTTCTTCAGTGTG-3′
COX2	Forward	5′-CCCAGCACTTCACGCATCAG-3′
	Reverse	5′-CGCTGTCTAGCCAGAGTTTC-3′
GAPDH	Forward	5′-GTCTCCTCTGACTTCAACAGCG-3′
	Reverse	5′-ACCACCCTGTTGCTGTAGCCAA-3′

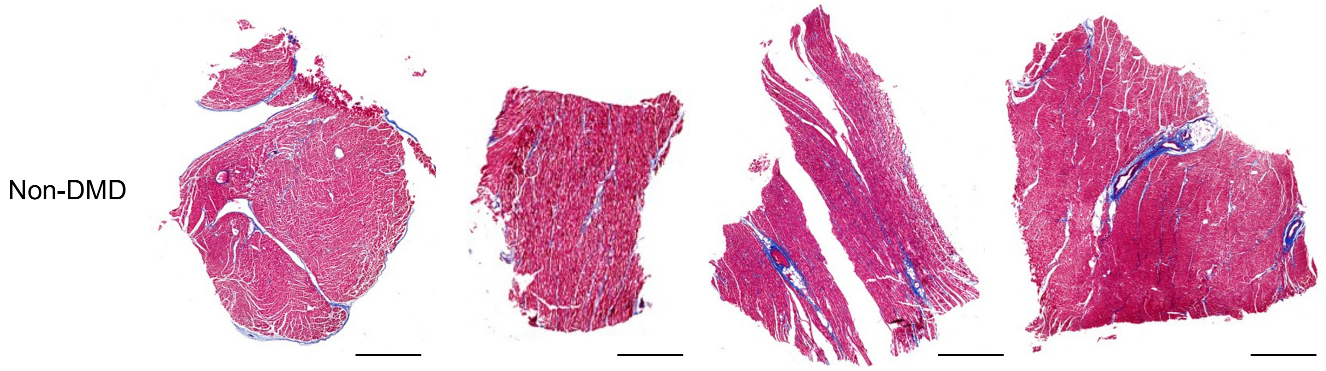

(**a**)

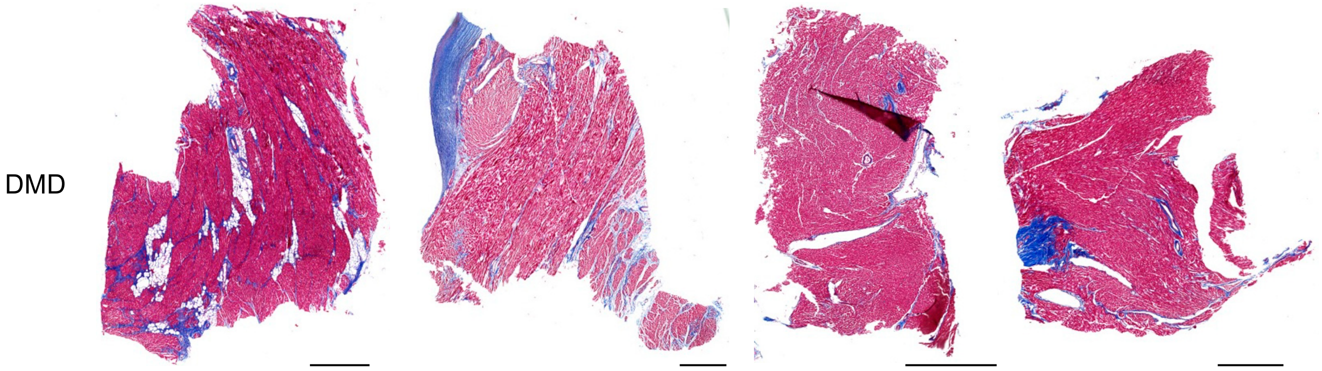

(**b**)

Supplementary Figure S1. Masson's trichrome stain images of autopsy myocardial specimens obtained from non-DMD (**a**) and DMD patients (**b**). The scale bar indicates 1,000 μm.

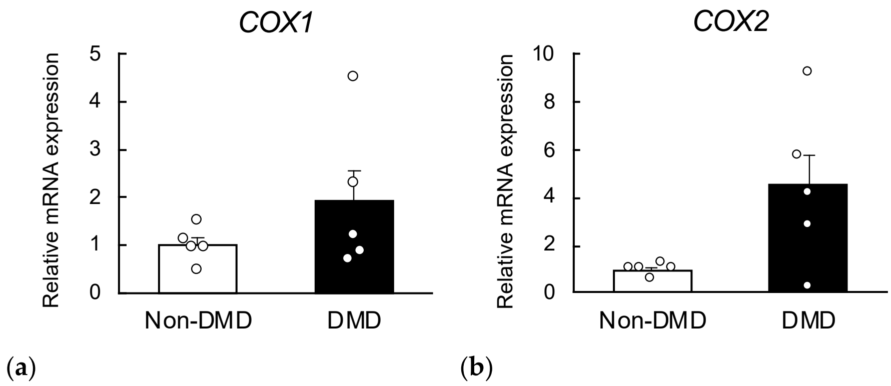

Supplementary Figure S2. Comparison of *COX1* (**a**) and *COX2* (**b**) mRNA expression levels in the autopsy myocardial specimens obtained from non-DMD and DMD patients. In all panels, each value represents the mean ± S.E. (n = 5). There were no significant differences between the two groups (unpaired Student's t test).

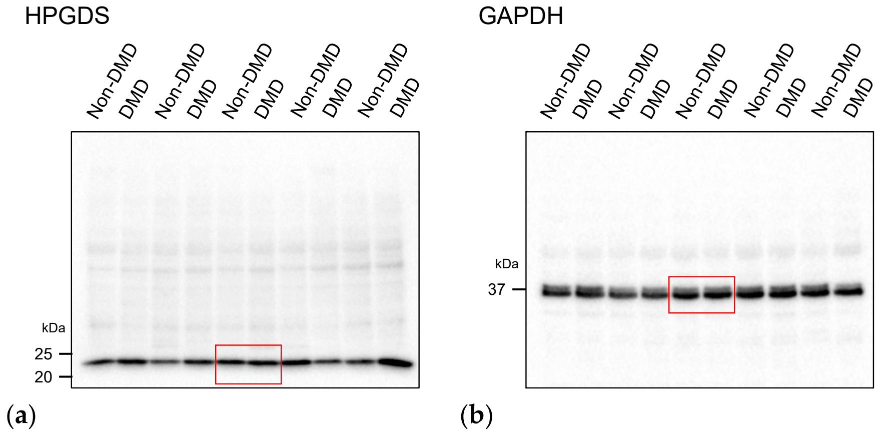

Supplementary Figure S3. Western blotting images of HPGDS (**a**) and GAPDH (**b**) proteins extracted from the autopsy myocardial specimens of non-DMD and DMD patients. The parts boxed in red were trimmed and used in Figure 2B.

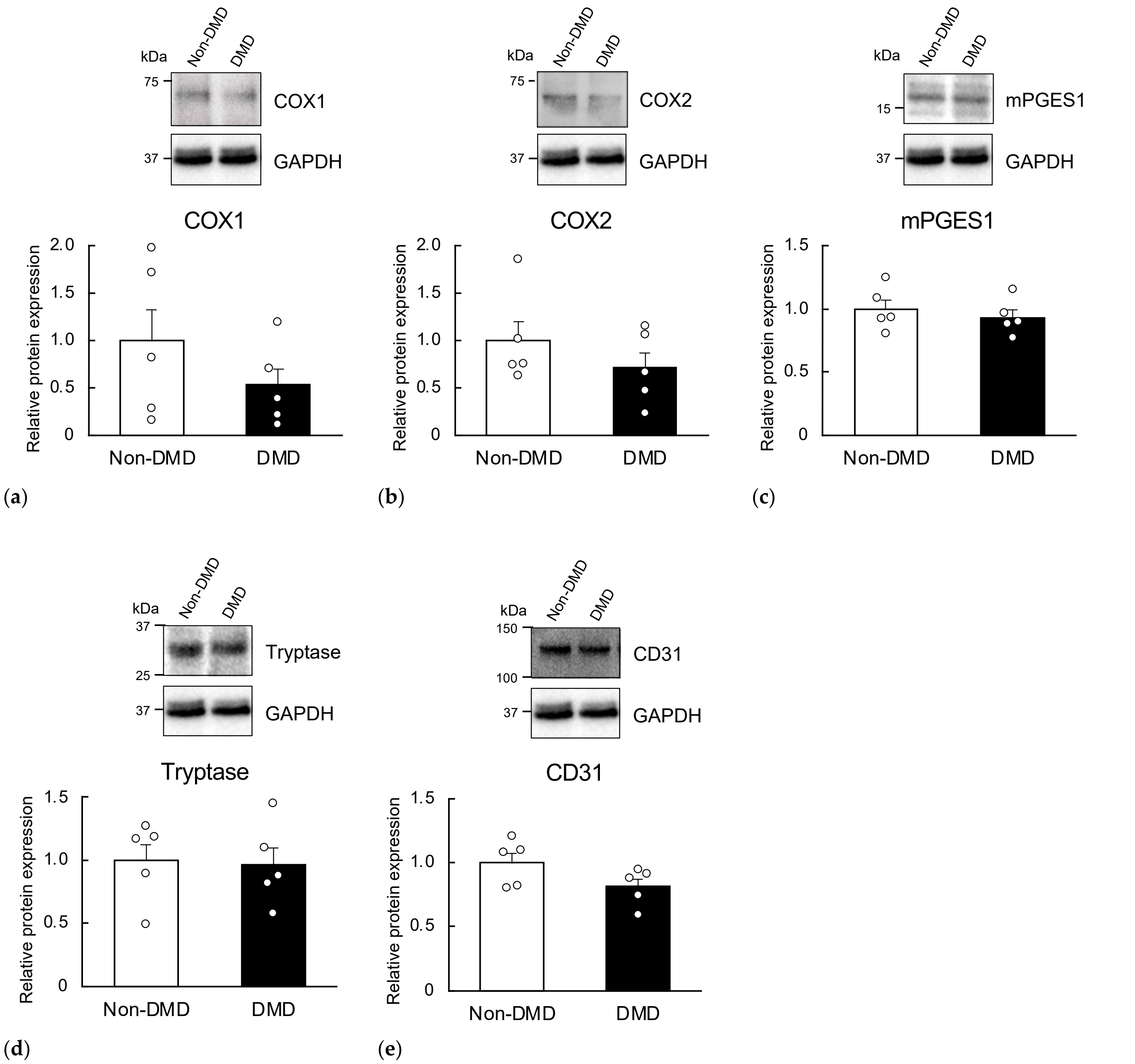

Supplementary Figure S4. Comparison of COX1 (**a**), COX2 (**b**), mPGES1 (**c**), Tryptase (**d**), and CD31 (**e**) protein expression levels in the autopsy myocardial specimens obtained from non-DMD and DMD patients. In all panels, each value is the mean ± S.E. (n = 5). There were no significant differences between the two groups (unpaired Student's t test).

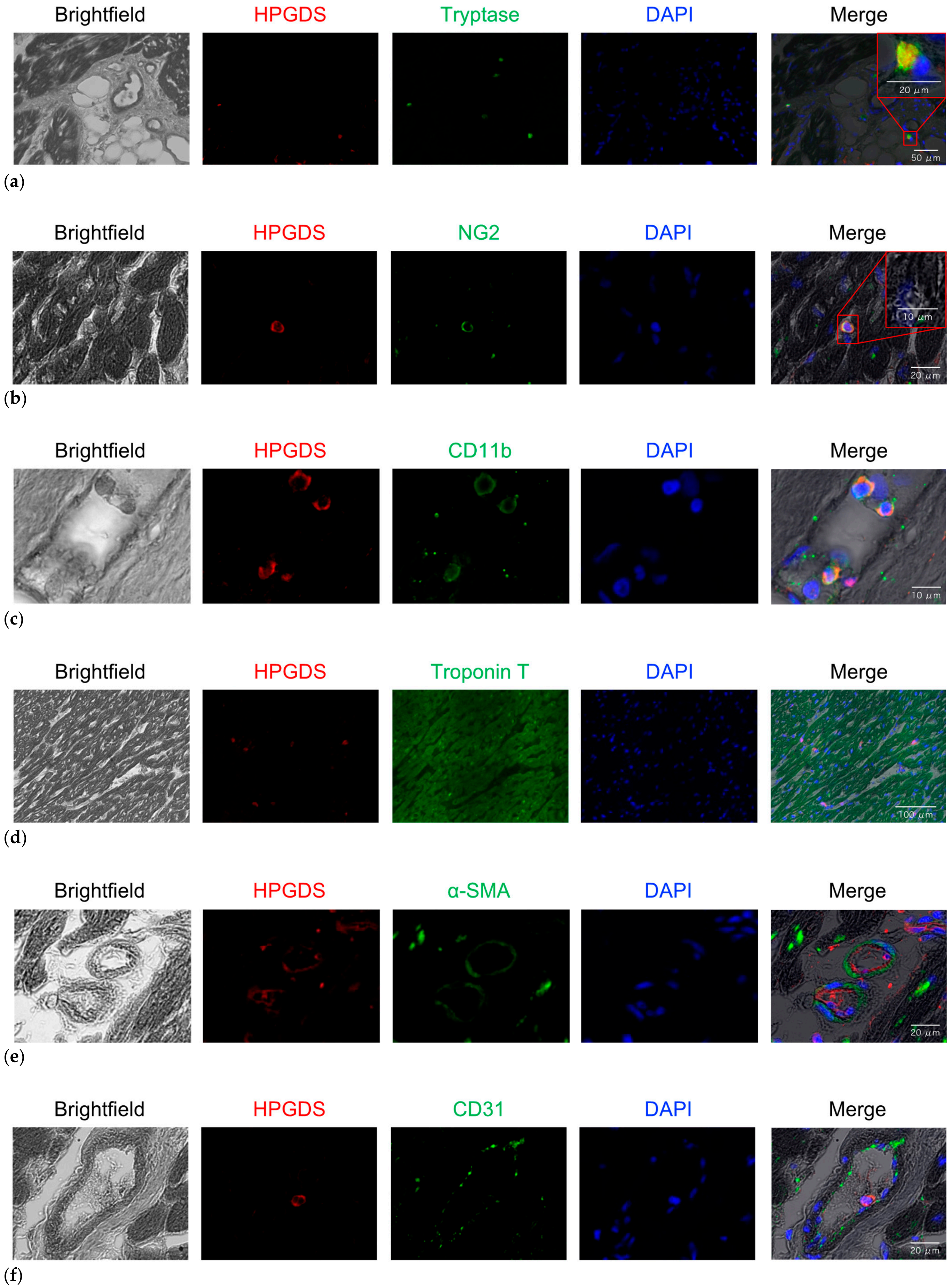
Brightfield
HPGDS
Tryptase
DAPI
Merge
20 μm
50 μm
(a)
Brightfield
HPGDS
NG2
DAPI
Merge
10 μm
20 μm
(b)
Brightfield
HPGDS
CD11b
DAPI
Merge
10 μm
(c)
Brightfield
HPGDS
Troponin T
DAPI
Merge
100 μm
(d)
Brightfield
HPGDS
α-SMA
DAPI
Merge
20 μm
(e)
Brightfield
HPGDS
CD31
DAPI
Merge
20 μm
(f)

Supplementary Figure S5. Representative images without superimposition of fluorescent immunostaining to identify HPGDS-expressing cells in the autopsy myocardial specimens obtained from DMD patients. All images have the same settings for HPGDS (red), DAPI (blue), and Bright-field, for green: Tryptase (**a**), NG2 (**b**), CD11b (**c**), Troponin T (**d**), α-SMA (**e**), and CD31 (**f**). Each merged image was used in Figure 2B. The scale bars are indicated on each image.

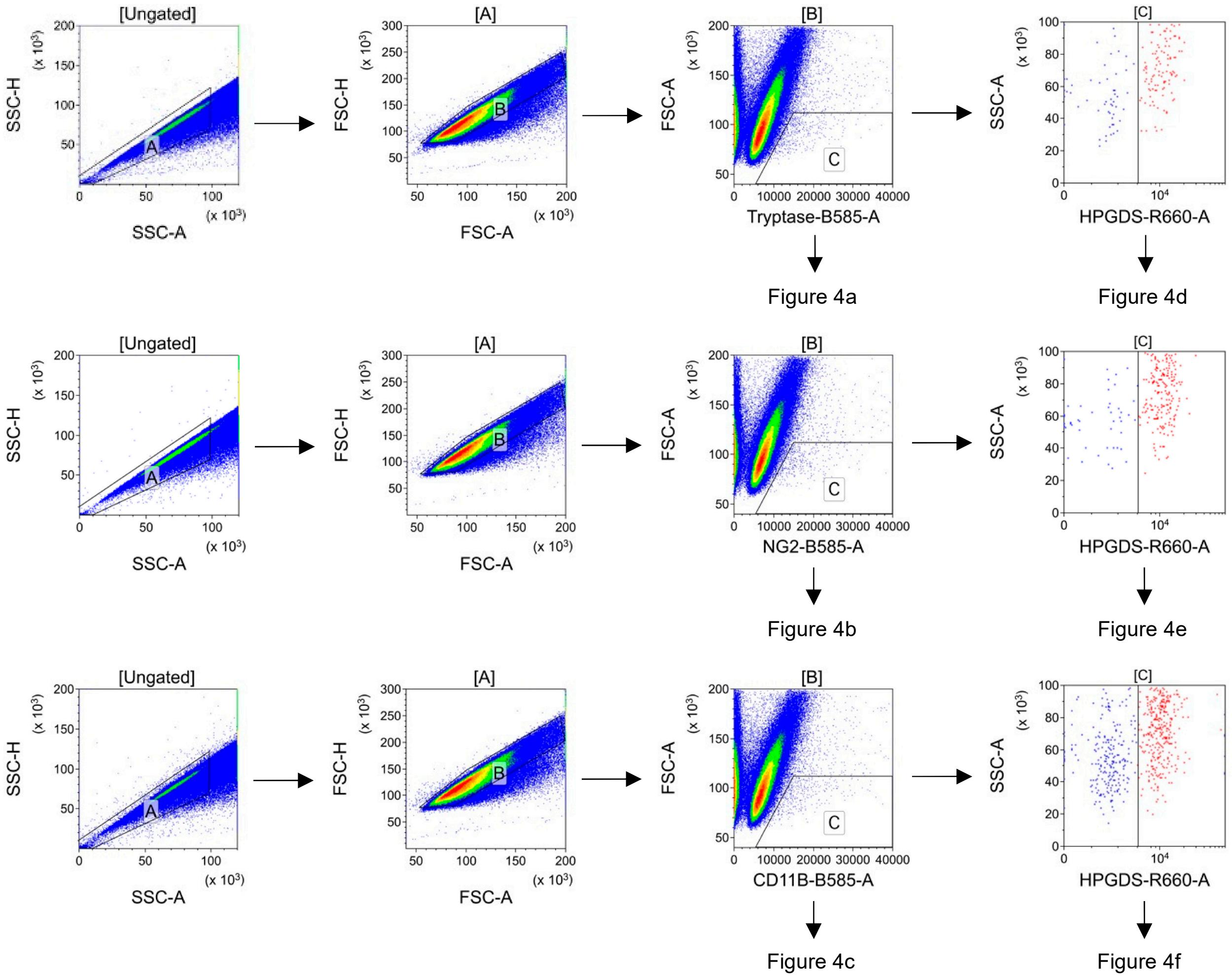

Supplementary Figure S6. Gating strategies used for flow cytometry analysis in Figure 4.
